# Bilateral Traumatic Frontal Hematoma Evacuation Using the Supraorbital Keyhole Approach: A Case Report Elucidating Surgical Technique

**DOI:** 10.7759/cureus.88822

**Published:** 2025-07-26

**Authors:** Alex Michel Daoud, Lorenna Filice, Jean Gonçalves De Oliveira, José Carlos E Veiga, João Luiz Vitorino Araujo

**Affiliations:** 1 Neurological Surgery, Irmandade da Santa Casa de Misericórdia de São Paulo, São Paulo, BRA; 2 Neurological Surgery, Hospital do Coração (HCor), São Paulo, BRA; 3 Neurological Surgery, Hospital São Camilo, São Paulo, BRA

**Keywords:** bilateral craniotomy, frontal hematoma, minimally invasive neurosurgery, supraorbital keyhole approach, traumatic brain injury

## Abstract

Bilateral frontal contusions following traumatic brain injury represent a neurosurgical emergency due to their association with intracranial hypertension and risk of herniation. Traditional craniotomies, although effective, often involve significant surgical trauma, prolonged recovery, and less favorable cosmetic outcomes. The supraorbital keyhole approach (SOKA) has emerged as a minimally invasive alternative, particularly for anterior cranial fossa lesions, but its use in extensive bilateral hematomas remains scarcely documented. We present the case of a 36-year-old man with bilateral frontal intraparenchymal hematomas who underwent hematoma evacuation via bilateral supraorbital craniotomies through eyebrow incisions. The procedure allowed near-total evacuation without significant retraction or neurovascular injury. Postoperative recovery was uneventful, with resolution of intracranial hypertension and favorable neurological and aesthetic outcomes. This case demonstrates the feasibility and safety of the bilateral SOKA for traumatic hematomas, suggesting its potential role as an alternative to traditional craniotomies in selected neurotrauma cases.

## Introduction

Traumatic brain injuries (TBIs) represent a significant global health burden, with contusions being one of the most common forms of intracranial damage following head trauma. Frontal contusions, in particular, are frequently observed in cases of deceleration injuries, such as those resulting from motor vehicle accidents or falls [1]. Bilateral frontal contusions pose a unique challenge, increasing the risk of secondary complications, including brain herniation and intracranial hypertension [2].

The surgical management of traumatic intracerebral hematomas (TICHs) arising from frontal contusions is crucial for preventing neurological deterioration. While conventional craniotomies can effectively be used for hematoma evacuation, they are often associated with longer operative times, greater blood loss, and increased brain retraction, which can exacerbate injury [3].

The supraorbital keyhole approach (SOKA) has emerged as a minimally invasive alternative, providing direct access to the frontal lobe through a small incision in the eyebrow. Originally developed for skull base tumor resection [4], this approach has expanded to include the evacuation of hematomas and contusions, offering significant benefits such as reduced surgical trauma, shorter recovery times, and improved cosmetic outcomes [5]. The versatility of this technique extends to trauma cases, including skull stab wounds and lesions involving the medial anterior cranial fossa, demonstrating its potential as a safe and effective minimally invasive approach [6]. While the SOKA is well established for skull base pathologies and localized hematomas, its application for extensive bilateral hematomas has not been widely documented. The available literature primarily reports unilateral approaches for smaller hematomas, making this case a rare example of its successful bilateral use. By minimizing brain retraction and preserving neurovascular structures, SOKA represents a valuable tool in the neurosurgical armamentarium, particularly for treating frontal contusions and hematomas in carefully selected patients. This case report highlights the successful application of the supraorbital approach for the evacuation of a bilateral frontal intraparenchymal hematoma, underscoring the technique’s efficacy. 

## Case presentation

A 36-year-old man was brought to the emergency department with a history of seizures and decreased consciousness. Upon examination, he presented with facial and scalp trauma stigmata, suggesting a possible head injury, though the circumstances and mechanism of trauma were unknown, as the event was not witnessed. On admission, his Glasgow Coma Scale score was 10 (E3 V3 M4).

**Figure 1:**
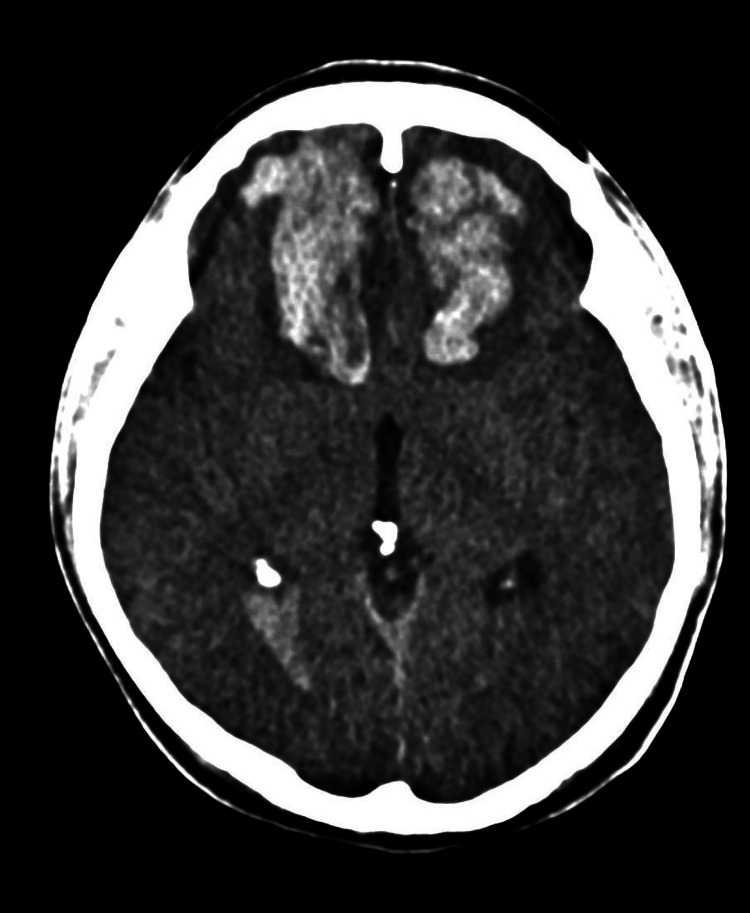
Head CT scan showing bilateral frontal intraparenchymal hematoma

A non-contrast brain computed tomography (CT) scan revealed acute intraparenchymal hemorrhages with surrounding edema, located bilaterally in the frontopolar and frontobasal regions. The hematomas obliterated the cerebrospinal fluid spaces in the area, particularly the frontal horns of the lateral ventricles. The left frontal hematoma measured 2.2 x 3.9 x 4.1 cm, with an estimated volume of 18.0 mL, while the right frontal hematoma measured 5.2 x 4.4 x 2.4 cm, with a volume of 28.0 mL (Figure 1). While a postictal state was considered, the patient’s progressive deterioration, imaging signs of compressed frontal horns and edema, and intraoperative evidence of brain swelling were consistent with intracranial hypertension. Thus, surgical intervention was indicated.

**Figure 2:**
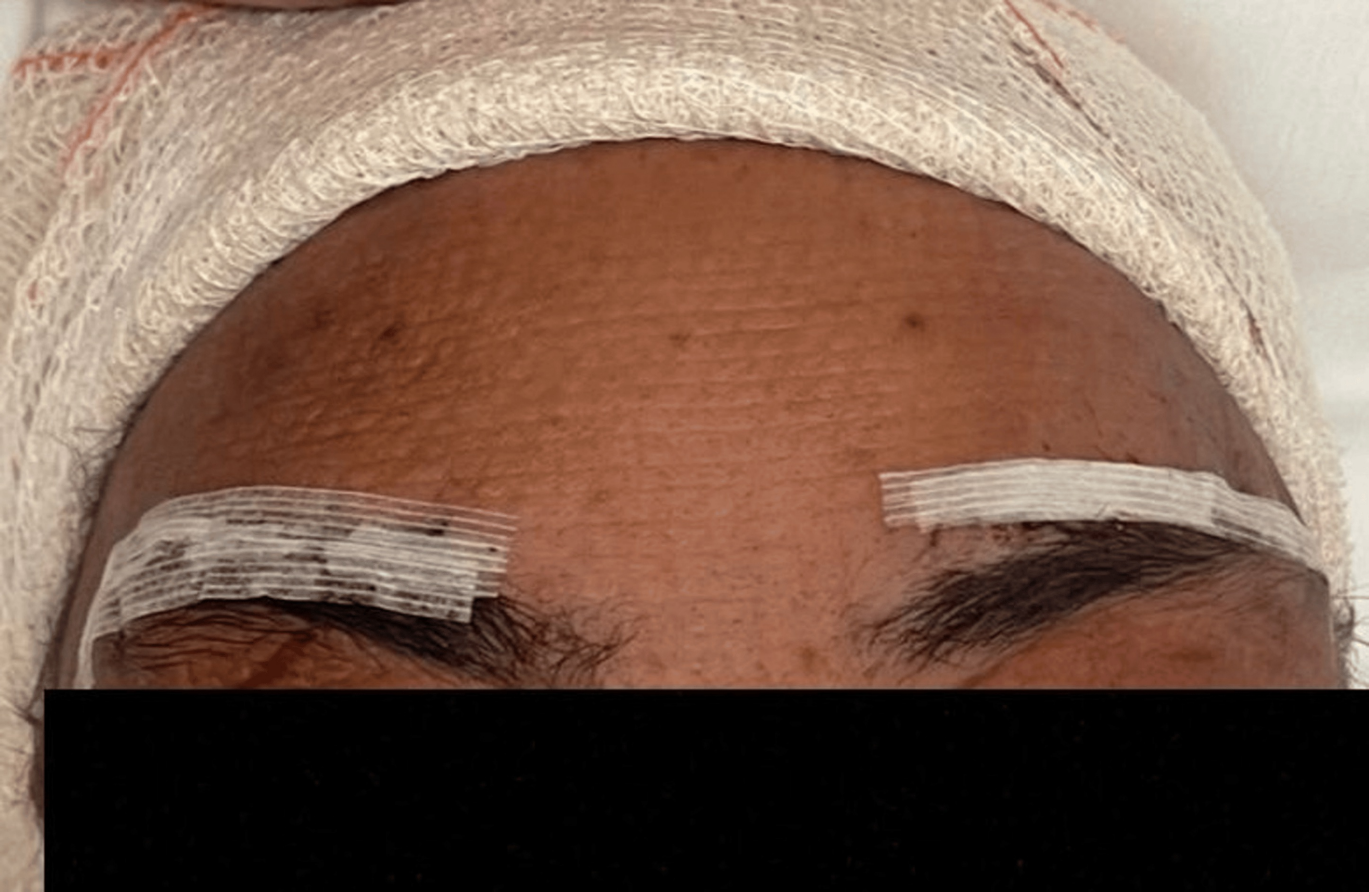
Dressing over the eyebrow incision in the immediate postoperative period

The patient underwent a bilateral supraorbital craniotomy through eyebrow incisions (Figure 2). He was placed in the supine position with his head in a neutral alignment. Near-total evacuation of the hematoma was achieved. No excessive bleeding was observed intraoperatively, and adjacent neurovascular structures were preserved.

**Figure 3:**
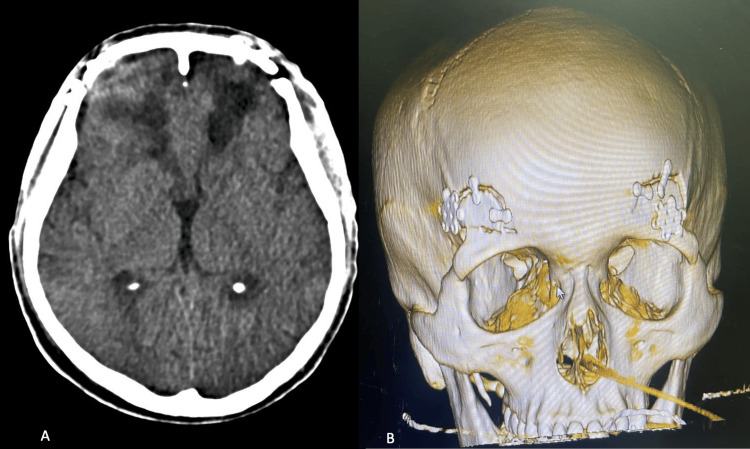
(A) Follow-up CT scan after evacuation of the hematoma, (B) 3D bone reconstruction of the bilateral supraorbital craniotomy

Postoperatively, the patient was extubated on the second day while still in the intensive care unit and was subsequently transferred to the general ward for neurofunctional recovery. A follow-up CT scan confirmed the near-complete evacuation of the hematoma and the resolution of intracranial hypertension (Figure 3).

On the 30th postoperative day, he developed late-onset post-traumatic hydrocephalus, which required the placement of a ventriculoperitoneal shunt.

The patient remained hospitalized for a total of 40 days. Upon discharge, he exhibited no focal neurological deficits but presented with mild behavioral disturbances, for which he was referred to psychotherapy. Significant improvement in his behavioral condition was noted during subsequent outpatient follow-ups.

Surgical technique

The patient was placed in the supine position with the head in a neutral and slightly extended position. The head was fixed using a three-pin Mayfield head holder, ensuring stability and allowing for sequential access to both sides of the frontal region. The head remained in a neutral position to facilitate symmetrical exposure.

A bilateral supraorbital incision was planned, starting with the right side. A 4-cm incision was made lateral to the supraorbital foramen, extending along the upper third of the eyebrow toward the frontal process of the zygomatic bone, carefully respecting the orbital rim. This step was then repeated on the left side, mirroring the incision to ensure symmetrical access.

After the skin incision on the right side, the skin flap was temporarily retracted with four stitches, exposing the frontal belly of the occipitofrontal muscle and the underlying orbicular and temporal muscles. The frontal muscle was incised parallel to the glabella, taking care to identify and preserve the supraorbital nerve to avoid iatrogenic injury. The temporal muscle was then detached from its bony insertion. The temporal muscle was retracted laterally with strong stitches, while the frontal muscle was elevated upwards. This entire process was subsequently performed on the left side, following the same steps to achieve bilateral exposure.

A single burr hole was created at the keyhole site on the right side, just below the superior temporal line. Using a high-speed craniotome, a straight line was cut from the burr hole in a medial direction, parallel to the glabella. The craniotomy was completed with a C-shaped cut, forming the bone flap. This sequence was then repeated on the left side, ensuring symmetrical bilateral access to the intracranial space.

Upon completion of the craniotomies, the dura mater on the right side was opened in a cruciate (X-shaped) fashion, allowing access to the underlying hematoma. Upon dural opening, the brain was found to be significantly swollen on both sides, indicating pronounced intracranial hypertension. The hematoma was gently evacuated under continuous irrigation. No brain retraction was necessary, as the cavity allowed for spontaneous decompression. This process was then performed on the left side, following the same technique for complete bilateral evacuation of the hematoma. No significant bleeding was observed during the procedure.

The closure process began on the right side, where the dura mater was sutured in a continuous fashion using 4-0 Prolene to ensure a watertight seal. The bone flap was repositioned and secured with titanium plates. The temporal muscle was reattached using 2-0 Vicryl sutures, and the skin was closed with a continuous 4-0 nylon suture. The same steps were meticulously repeated on the left side to ensure symmetry and optimal cosmetic results.

Intraoperatively, slight adhesion of the hematoma to the brain parenchyma was noted bilaterally but did not present significant technical difficulties. The surgery proceeded without complications, and the patient tolerated the procedure well.

## Discussion

This case highlights the efficacy and expanding indications of the SOKA in managing bilateral frontal hematomas. Initially developed for skull base tumors and aneurysm surgeries, SOKA has demonstrated increasing value in trauma neurosurgery by providing direct access to the anterior cranial fossa with minimal invasiveness. The successful application of this technique for bilateral hematoma evacuation reinforces emerging evidence supporting the use of minimally invasive approaches in TBI management [1,2].

One of the key aspects of this case was the significant brain swelling observed upon dural opening, reflecting severe intracranial hypertension. This highlights the importance of early surgical decompression in cases of bilateral frontal hematomas to prevent secondary brain injury and herniation [3]. By preserving neurovascular structures and limiting parenchymal manipulation, SOKA contributes to better long-term recovery in patients with TICHs [4].

The cosmetic and functional advantages of SOKA compared to traditional craniotomies are well documented. Studies have shown that supraorbital craniotomies result in shorter hospital stays, less postoperative pain, and lower rates of infection compared to larger frontal or pterional craniotomies [5]. In addition, the use of small incisions along natural facial lines, such as the eyebrow, contributes to superior cosmetic outcomes, addressing concerns related to visible scarring and deformity [7]. Reports have also demonstrated that the medial supraorbital craniotomy offers the advantage of minimizing manipulation of the temporal muscle, reducing the risk of injury to the frontotemporal branch of the facial nerve and providing direct access to the anterior cranial fossa [6]. This approach is particularly advantageous in younger patients or those for whom aesthetic considerations are paramount. Although SOKA is effective for localized hematomas and anterior cranial lesions, its application in deeper or multilobar hematomas may be limited. The technique relies on achieving adequate exposure through small craniotomies, which may not be suitable for extensive hemorrhages involving deeper brain structures or those extending into the posterior cranial fossa [8]. Additionally, the procedure's success is influenced by the surgeon’s experience with keyhole techniques, highlighting the need for specialized training and skill development [9-11].

To the best of our knowledge, the existing literature predominantly describes the use of the unilateral supraorbital keyhole approach for the evacuation of smaller hematomas or ipsilateral hematomas. However, we found no documented cases using a bilateral supraorbital approach for the evacuation of such an extensive bilateral hematoma. This exceptional case underscores the feasibility of this technique in selected trauma patients and expands its potential indications in neurotrauma surgery.

## Conclusions

This case contributes to a growing body of literature advocating for the broader application of SOKA in neurotrauma. While traditional craniotomies remain the gold standard for large hematomas, expanding the indications for minimally invasive techniques may improve patient outcomes, reduce recovery times, and minimize surgical morbidity. Future cohorts studies are necessary to validate the long-term efficacy of SOKA in trauma cases and establish standardized guidelines for its use.
